# Comparing Rates of Change in Moderate to Advanced Glaucoma: Retinal Nerve Fiber Layer Versus Bruch Membrane Opening-Minimum Rim Width

**DOI:** 10.1016/j.ajo.2023.05.003

**Published:** 2023-05-06

**Authors:** LYNN SHI, MASSOOD MOHAMMADI, VAHID MOHAMMADZADEH, ERICA SU, ROBERT E. WEISS, JOSEPH CAPRIOLI, KOUROS NOURI-MAHDAVI

**Affiliations:** Glaucoma Division, Stein Eye Institute, David Geffen School of Medicine, University of California Los Angeles, Los Angeles, CA, USA (L.S, M.M, V.M, J.C, K.N-M); Department of Biostatistics, Fielding School of Public Health, University of California Los Angeles, Los Angeles, CA, USA (E.S, R.E.W)

## Abstract

**PURPOSE:**

To compare rates of change (RoC) of peripapillary retinal nerve fiber layer (RNFL) and Bruch membrane opening-based minimum rim width (BMO-MRW) thickness in moderate-to-advanced glaucoma.

**DESIGN:**

Prospective cohort study.

**METHODS:**

Longitudinal optical coherence tomography (OCT) optic nerve head volume scans of 113 eyes of 113 glaucoma patients with moderate-to-advanced or central damage were exported. This study estimated and compared global and sectoral RoC with linear mixed effects models and simple linear regression (SLR) of RNFL and BMO-MRW thickness. Permutation analyses were used to test significance of RoC in the SLR model. It also compared longitudinal signal-to-noise ratios (LSNR) defined as RoC divided by residual standard deviation (SD) between the two groups.

**RESULTS:**

Mean (SD) follow-up and median (IQR) OCT scan sessions were 5.2 (1.3) years and 10 (8–11), respectively. Baseline average (SD) visual field mean deviation was −9.2 (5.8) dB. Based on SLR, a higher proportion of significant negative RNFL RoC was observed compared to BMO-MRW in the inferotemporal (35% vs 20%; *P* = .015) and inferonasal (42% vs 17%; *P* < .001) sectors. Permutation analyses also demonstrated a higher proportion of worsening RNFL RoC than BMO-MRW in the inferotemporal (*P* = .026) and inferonasal (*P* < .001) sectors along with overall lower positive RoC. Longitudinal signal-to-noise ratios for RNFL were significantly more negative than for BMO-MRW globally, and in the inferotemporal, inferonasal, and superonasal sectors (*P* ≤ .01).

**CONCLUSIONS:**

Longitudinal RNFL OCT measurements are more likely to detect structural change and demonstrate better LSNR compared with BMO-MRW in eyes with central or moderate-to-advanced glaucoma damage at baseline.

## INTRODUCTION

Detection of progression is a crucial task in glaucoma management and, if carried out properly, it can lead to timely escalation of the intensity of glaucoma treatment and slowing of glaucoma damage.^1, 2^ Optical coherence tomography (OCT) is a powerful tool for identifying glaucoma damage and detecting structural changes over time. It allows quantitative and objective measurements of the retinal nerve fiber layer (RNFL), neuroretinal rim, and macula, and has become an essential clinical tool for assessing glaucoma deterioration.^1^

Measurement of rates of change (RoC) in glaucoma has largely focused on the circumpapillary RNFL, which is the most widely used metric in the clinical setting.^3–7^ The Bruch membrane opening-based minimum rim width (BMO-MRW) was introduced by Chauhan and Burgoyne^8^ as a superior metric for assessing the retinal ganglion cell axonal complement at the level of the neuroretinal rim; it measures the shortest distance between the inner opening of the Bruch membrane opening (BMO) and the internal limiting membrane. An early study reported that the BMO-MRW thickness showed a stronger structure-function relationship with visual fields than the circumpapillary RNFL.^9^ However, a subsequent study found that structure-function correlations were weaker for BMO-MRW compared with RNFL thickness.^10^ While early diagnosis of glaucoma is essential, monitoring eyes with suspected or established glaucoma for subsequent change is a crucial task; this is particularly challenging in eyes with moderate-to-advanced glaucoma, in which the visual fields display higher long-term fluctuation.^11^

There are few longitudinal studies comparing the performance of the neuroretinal rim as captured with BMO-MRW thickness and the RNFL rates of change for detection of progression in glaucoma patients.^12, 13^ The results of these investigations suggest equivalent performance of these parameters in identifying progression at different stages of glaucoma.

The current study aimed to estimate and compare rates of change of peripapillary RNFL and BMO-MRW thickness in glaucoma eyes with central or moderate-to-advanced damage at baseline. It compared longitudinal signal-to-noise ratios (LSNR) and the proportion of worsening and improving eyes based on global and sectoral RNFL and BMO-MRW rates of change.

## METHODS

### STUDY SAMPLE:

Data from 113 glaucoma eyes (113 patients) from the Advanced Glaucoma Progression Study—an ongoing, prospective, longitudinal study at the University of California, Los Angeles—were analysed.^14^ Institutional Review Board approval was obtained for this study. The study adhered to the tenets of the Declaration of Helsinki and conformed to the Health Insurance Portability and Accountability Act policies. All patients provided written informed consent at the time of enrollment in the study. The enrolled eyes met the following inclusion criteria: a) clinical diagnosis of primary open-angle glaucoma, pseudoexfoliative glaucoma, pigmentary glaucoma, or primary angle-closure glaucoma; b) evidence of either central damage on 24–2 visual field, defined as two or more points within the central 10° with *P* < .05 on the pattern deviation plot or a visual field mean deviation (MD) of −6 dB or worse. The following were exclusion criteria: baseline age < 40 years or > 80 years, best corrected visual acuity worse than 20/50, refractive error exceeding 8 diopters (D) of sphere or 3 D of cylinder, and any significant retinal or neurological disease potentially affecting OCT measurements. Study eyes had no other ocular pathology at baseline and underwent clinical exams, imaging, and visual field testing approximately every 6 months. All eyes had at least 18 months of follow-up and at least three OCT imaging sessions, and the range of follow-up duration and scan numbers were 1.6 to 6.7 years and 3 to 22, respectively.

### OPTIC NERVE HEAD OPTICAL COHERANCE TOMOGRAPHY IMAGING:

The Spectralis spectral-domain OCT (SD-OCT) (Heidelberg Engineering) was used to obtain optic nerve head (ONH) volume scans. The Glaucoma Module Premium Edition (GMPE), a proprietary software of the Spectralis OCT, acquires three circumpapillary RNFL scans 12°, 14°, and 17° in diameter (corresponding to 3.5 mm, 4.1 mm, and 4.7 mm in an emmetropic eye) centered on the BMO. Only data from the 12° measurement circle were used for this study. Additionally, each scan acquires 24 radial line scans centered on the BMO centroid, which are subsequently used to measure the BMO-MRW thickness at 24 locations around the BMO. The software automatically segments the RNFL and BMO-MRW. Images were reviewed for centration, segmentation errors, and image artifacts. Any obvious segmentation errors were manually corrected with the SD-OCT device’s built-in software. If any individual volume scans were of inadequate quality or showed poor segmentation that could not be rectified, both RNFL and BMO-MRW data for that session were excluded from the analyses. A low-quality OCT image was defined as quality factor < 15, presence of > 10% missing data or inadequate segmentation, or any artifacts. After segmentation, the RNFL and BMO-MRW measurements were exported to a personal computer. In addition to global or average RNFL and BMO-MRW thickness measurements, Spectralis OCT provides the sectoral thickness measurements in six sectors as follows: temporal (311–40°), temporal superior (41–80°), nasal superior (81–120°), nasal (121–230°), nasal inferior (231–270°), and temporal inferior (271–310°). The junction of the measurement circles and the axis connecting the foveal center to the BMO centroid (Fo-BMO axis) is the starting point for scanning and definition of sectors by GMPE software.

### STATISTICAL ANALYSES:

Global and sectoral RoC were estimated with simple linear regression (SLR) of RNFL thickness and BMO-MRW against time and with a longitudinal random intercept and slope liner mixed effects model. Based on SLR, the proportions of worsening or improving RoC (negative or positive RoC with *P* < .05) for global and sectoral thicknesses of RNFL and BMO-MRW were calculated and compared between the two measures with McNemar’s test. Permutation analyses were used to control the Type I error for detection of change with the 2.5%ile or 97.5%ile cutoff points used to reject the null hypothesis of no change in favor of significant negative or significant positive rate of change, respectively.^15–17^

Simple linear regression results in estimates that are un-biased for individual slopes on average; however, SLR does not have a way of estimating population slopes or variances of slopes across individuals. Thus, rates of change, globally and in sectors, were estimated with a random intercept and slope linear mixed model providing estimates and standard errors (SE) of population intercepts, population slopes, and intercept and slope random effect variances.

Given the different measurement range of the BMO-MRW and RNFL thickness measurements, LSNR were calculated for BMO-MRW and RNFL by dividing the SLR RoC by the SLR residual standard deviation, and the LSNRs of BMO-MRW and RNFL were compared with a Wilcoxon signed rank test.^18^

To assess the linearity of the longitudinal trend for RNFL and BMO-MRW thicknesses, the profile plots for all subjects and all sectors were inspected as part of the data analysis procedures. Empirical summary plots of empirical residuals for all sectors were also inspected. These plots first centered observations around the subject (or subject/sector) average thickness during follow-up, creating within subject empirical residuals. Empirical residuals were then averaged across subjects for a given sector within regular time windows (0–3 months, 3–9 months, and so on for continuing regular 6-month intervals) and mean empirical residuals plus or minus twice the SE of the mean were plotted.

Summary data are presented as mean (SD or SE) for continuous variables, median (range [minimum, maximum] or interquartile range [IQR]) for count variables and counts and percentages for categorical variables. A *P* < .05 was considered as statistically significant. The statistical analyses were performed with R (R version 4.1.0; www.R-project.org).

## RESULTS

Table 1 summarizes baseline clinical and demographic characteristics of the study patients. One hundred thirteen eyes from 113 glaucoma patients were included in the final analysis. The average (SD) age of patients was 67.7 (8.4) years. The mean (SD) follow-up period was 5.2 (1.3) years. The average (SD) baseline 24–2 visual field mean deviation was −9.2 (5.8) dB.

### RETINAL NERVE FIBER LAYER AND BRUCH MEMBRANE OPENING-MINIMUM RIM WIDTH BASELINE MEASUREMENTS AND RATES OF CHANGE:

**FIGURE 1.**
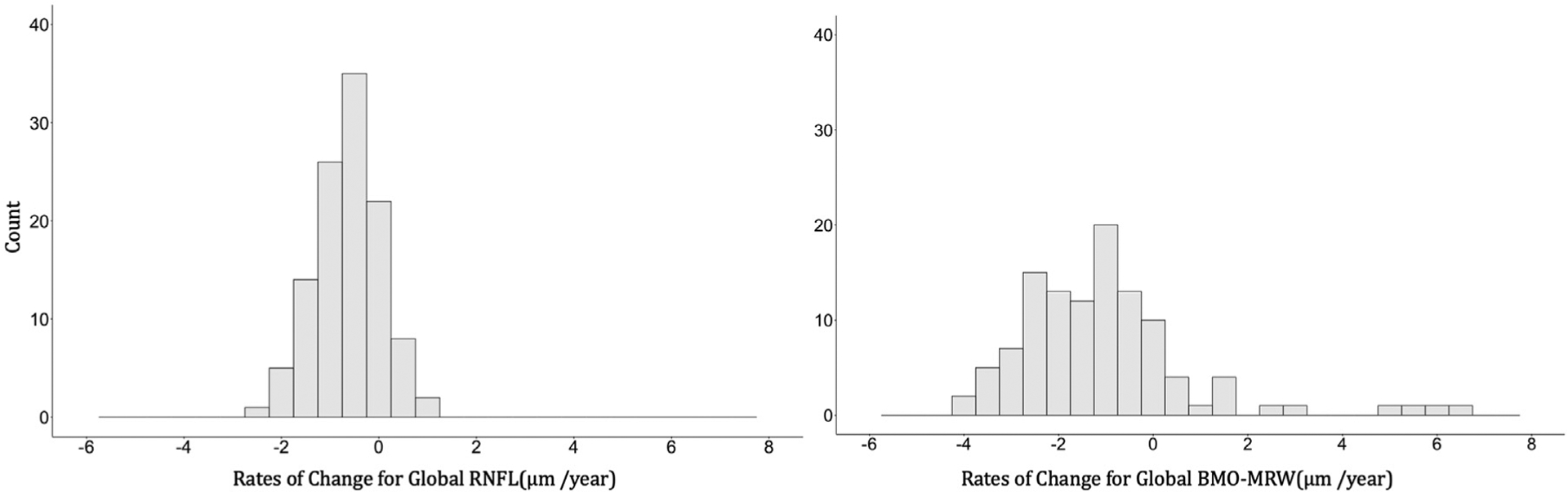
Histograms of the estimated rates of change of global retinal nerve fiber layer (RNFL) and Bruch membrane opening-minimum rim width (BMO-MRW) thickness from linear mixed effects model (LMM).

Estimates of global and sectoral BMO-MRW and RNFL thickness population intercepts (i.e., estimated baseline thickness), SD of the subject intercepts, population RoC, and SD of the subject RoCs are presented in Table 2 and the distributions of individual-eye RoCs for global RNFL and BMO-MRW are depicted in Figure 1. For global RNFL thickness, the population RoC (SE) was −0.64 (0.08) μm/year and the population SD (SE) of the RoCs was 0.75 (0.06). For global BMO-MRW, the population RoC (SE) and the population SD (SE) of the RoC were −1.1 (0.27) μm/year and 2.39 (0.23), respectively. For RNFL, the fastest rate of decline was observed in the inferotemporal sector, followed by the inferonasal and superonasal sectors. The rate of change for BMO-MRW was fastest in the temporal sector, followed by the superonasal and superotemporal sectors.

### COMPARISON OF THE PROPORTIONS OF EYES WITH WORSENING/IMPROVING RATE OF CHANGE:

**FIGURE 2.**
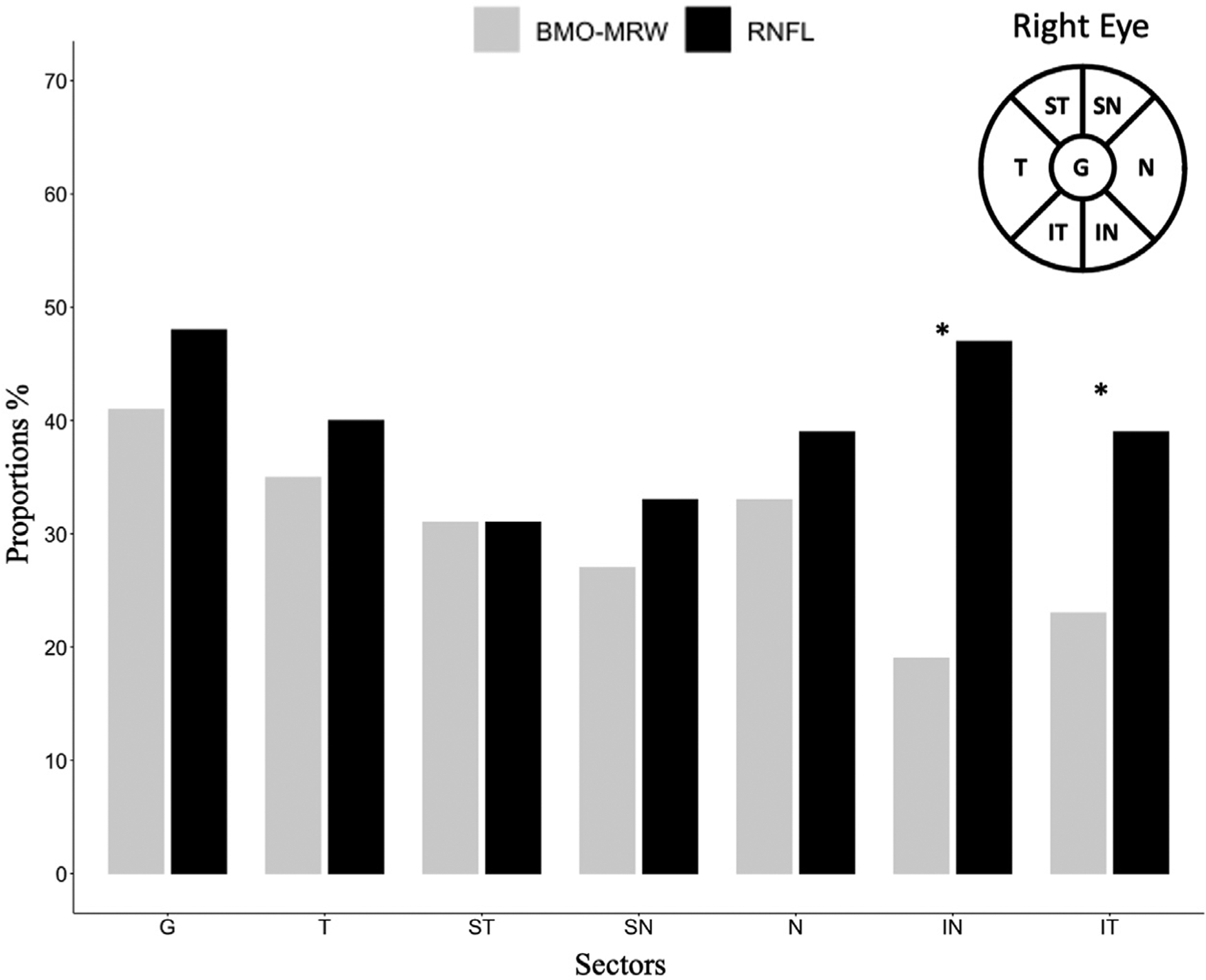
Bar graph of the proportion of significant (*P* < .05) worsening rates of change for retinal nerve fiber layer (RNFL) and Bruch membrane opening-minimum rim width (BMO-MRW), globally and in six sectors. *P < .05 from McNemar’s test comparing the corresponding RNFL and BMO-MRW proportions. G = global, T = temporal, ST = superior-temporal, SN = superior-nasal, N = nasal, IN = inferior-nasal, IT = inferior-temporal circumpapillary RNFL and BMO-MRW OCT sectors.

In the SLR models, a higher proportion of significant negative RoCs was observed for RNFL compared with BMO-MRW in the inferotemporal (35% vs 20%; *P* = .015) and inferonasal (42% vs 17%; *P* < .001) sectors. The proportion of improving sectors was not significantly different between the two measures, except in the inferotemporal sector (2.6% vs 9.7% for RNFL vs BMO-MRW, respectively; *P* = .04). Bar charts in Figure 2 display the proportion of significantly worsening RoC globally and by sectors for RNFL and BMO-MRW thickness. The proportion of worsening and improving RoC for RNFL and BMO-MRW based on permutation analysis are displayed in Table 3. Permutation analysis demonstrated a higher proportion of worsening RNFL rates than BMO-MRW in the inferotemporal (*P* = .026) and inferonasal (*P* < .001) sectors along with overall lower positive RoC.

### COMPARISON OF RETINAL NERVE FIBER LAYER AND BRUCH MEMBRANE OPENING-MINIMUM RIM WIDTH LONGITUDINAL SIGNAL-TO-NOISE RATIOS:

**FIGURE 3.**
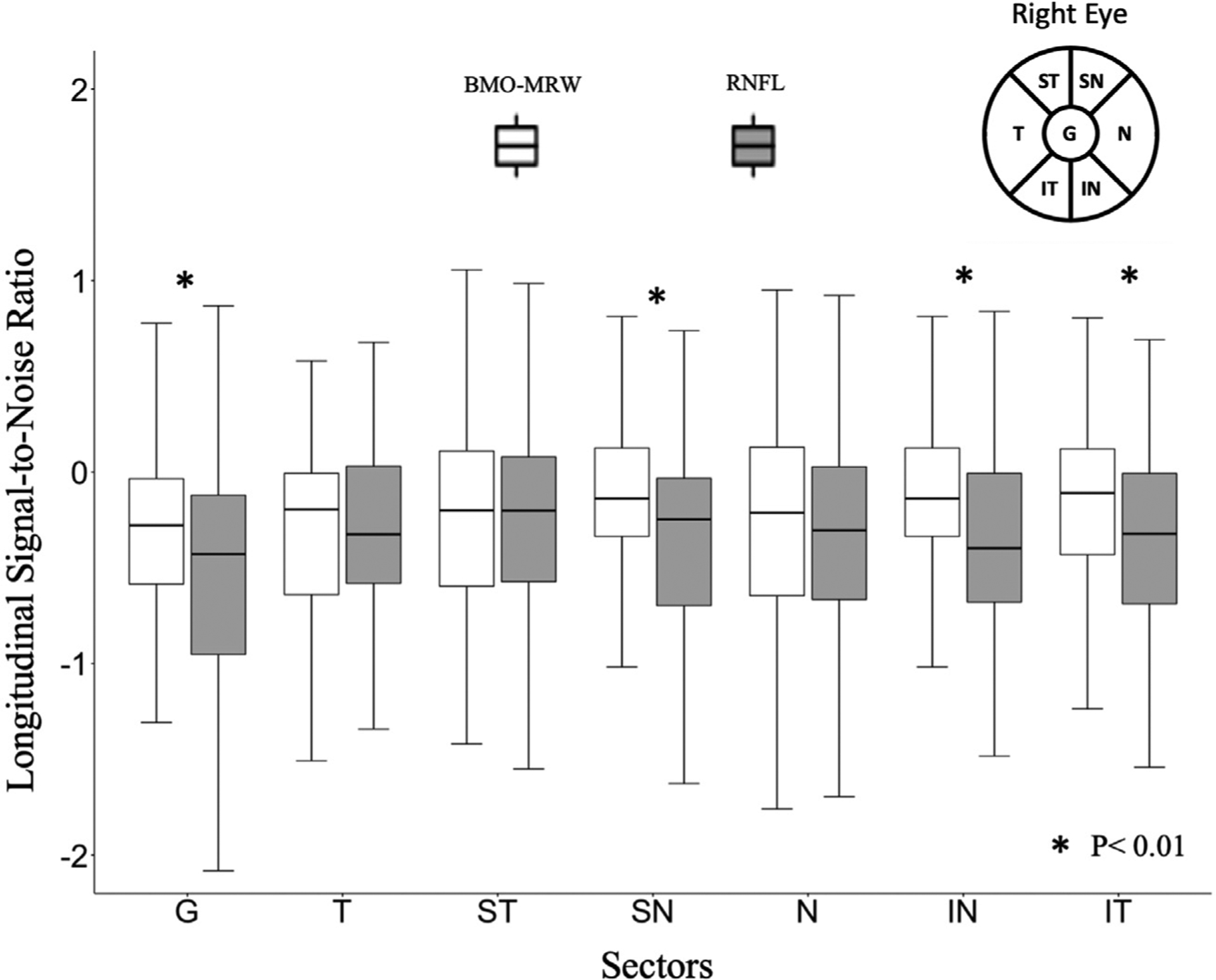
The distribution of global and sectoral longitudinal signal to noise ratio (LSNR) for retinal nerve fiber layer (RNFL) and Bruch membrane opening-minimum rim width (BMO-MRW) measures in the study cohort; LSNR was calculated by dividing the estimated slopes by residual SD from simple linear regression. Sectors with a significant difference between the RNFL and BMO-MRW rates are indicated with *. In all such sectors a more negative LSNR was observed for RNFL compared with BMO-MRW. Wilcoxon signed rank test was used to compare the pairwise rates of change. G = global, T = temporal, ST = superior-temporal, SN = superior-nasal, N = nasal, IN = inferior-nasal, IT = inferior-temporal circumpapillary RNFL and BMO-MRW OCT sectors.

The RNFL LSNR were significantly lower (more negative) than those of BMO-MRW globally (*P* = .007) and in the inferotemporal (*P* < .001), superonasal (*P* < .001), and inferonasal (*P* < .001) sectors. The distribution of the global and sectoral LSNR values for RNFL and BMO-MRW thickness is shown in Figure 3.

## DISCUSSION

Since the introduction of BMO-MRW as the preferred measure for assessing the neuroretinal rim with OCT in glaucoma, there have been studies exploring the utility of this measurement for detecting glaucoma, evaluating structure-function relationships, and identifying structural glaucoma deterioration.^10, 19–21^ The latter task is highly clinically relevant, as timely detection of structural changes in glaucoma patients can pave the way for an earlier change in treatment and prevention of glaucoma progression.

This study explored the longitudinal changes in RNFL and BMO-MRW thickness in a group of patients with central or moderate-to-advanced glaucomatous damage. The results suggest that RNFL thickness is more likely than BMO-MRW to reveal a declining trend over time in this group of eyes. Globally and within inferior and superonasal sectors, the LSNR for RNFL were more negative than those of BMO-MRW, meaning that the information within longitudinal RNFL thickness measurements is less noisy compared with that of BMO-MRW. We used permutation analysis to set the Type I error for detection of change at 0.05, which again showed a higher incidence of RNFL thinning in the inferotemporal and inferonasal sectors compared with BMO-MRW thickness. Although the average visual field MD was −9.2 dB in this study cohort, the inferotemporal and inferonasal sectors still displayed the highest proportion of significantly worsening rates of change, which suggests that these two sectors had not reached their measurement floor in many eyes with central or moderate-to-advanced damage, as defined in this study, and that additional structural progression may be detected in such eyes in these sectors.

Structural glaucomatous changes are thought to include active remodeling of connective tissues.^9^ In a group of experimental glaucoma eyes in nonhuman primates, He and associates^5^ found that changes in the OCT ONH measures including BMO-MRW consistently preceded RNFL thickness changes. The authors hypothesized that in early glaucoma, RNFL may be less affected by conformational changes, whereas BMO-MRW may be more sensitive to such changes. However, current clinical data have not provided strong evidence for the superiority of BMO-MRW over RNFL in identifying the earliest evidence of glaucoma damage. In a study including normal, preperimetric, and perimetric glaucoma subjects, Gmeiner and associates^12^ suggested that BMO-MRW and RNFL thickness were comparable for discriminating preperimetric and perimetric glaucoma eyes form normal subjects.

Few longitudinal clinical studies have compared the performance of RNFL and BMO-MRW thickness measures for detecting glaucoma progression.^20, 22^ Gardiner and associates^20^ investigated a cohort mostly comprising patients with very early glaucoma (average visual field MD of −1.2 dB), and reported that RNFL rates had a better signal-to-noise ratio compared with BMO-MRW and minimum rim area rates of change for detecting progression. In another study, Staag and Medeiros^22^ demonstrated that RNFL thickness may be better than BMO-MRW for detecting early disease in a longitudinal study of suspected glaucoma patients with a mean baseline visual field MD of −0.2 dB.^22^ In the latter study, the global RNFL sensitivity was 60% for detecting change (at 95% specificity) vs 40% for the global BMO-MRW.^22^

There are no longitudinal studies on the utility of RNFL and BMO-MRW rates of change and their longitudinal variability in moderated-to-advanced stages of glaucoma. This subgroup of glaucoma patients is of particular importance to clinicians, as they are at the highest risk of functional visual loss and blindness in case of disease progression. The current study provides evidence that the RNFL is more likely to show significant change compared with BMO-MRW in this group of patients, either globally or in some sectors. Based on these findings, this can be explained by the higher variability of the BMO-MRW measurements (see below).

Efficient monitoring and detection of glaucoma progression rely on a high signal-to-noise ratio and the magnitude of detectable change needs to be greater than longitudinal variability. Given the different ranges of RNFL and BMO-MRW measurements, this study used the method introduced by Gardiner and associates^18^ by dividing the rates of change by the SD of the regression residuals to define LSNR. We found that RNFL measurements had a lower (more negative) LSNR than BMO-MRW globally and in more sectors, confirming RNFL as the preferred measure for monitoring progression in eyes with central or moderate-to-advanced glaucoma damage.

There are possible explanations as to why BMO-MRW thickness, as it is currently estimated and provided by Spectralis OCT, may not perform as well as the RNFL thickness for detecting structural change in glaucoma. Gardiner and associates^20^ hypothesized that the higher variability of BMO-MRW stems from ONH conformational change and remodeling during the disease process and the impact from intraocular pressure on the ONH structure. Unlike the RNFL measurement circles, the radial scans based on which BMO-MRW thickness is estimated are not registered to the baseline scans and therefore, at least theoretically, there is more opportunity for measurement noise to be encountered over consecutive imaging sessions. Identifying the tip of the BMO on each ONH radial scan is a more challenging task to automate compared with segmentation of the RNFL, although the Spectralis OCT automated algorithm has been optimized for this task and based on current experience performs very well.^23, 24^ A check of our current correction procedure showed that manual correction for segmentation errors was performed for 19% of RNFL scans compared with 43% of BMO-MRW scans. Rezapour and associates^23^ found that in non-highly myopic eyes, indiscernible BMO was mostly seen in the nasal and temporal quadrants, and vessel shadowing caused particular difficulty in the nasal quadrant for BMO tip identification.^23^ However, most progressive glaucomatous changes occur in the superior and inferior regions of the ONH; the current findings also suggest that this may not have been a major confounding factor given that RNFL changes in the nasal region were not prominent.

A caveat of this study is the assumption that longitudinal change is linear over time. This is not definitively known and the relationship between RNFL and BMO-MRW has been suggested to be nonlinear.^25^ A review of the spaghetti plots (i.e., the longitudinal trends of thickness measurements within individual sectors or globally within eyes) failed to reveal obvious departures from linearity (Supplemental Figures 1 and 2).

The utility of a structural measure for detection of change is partly determined by its LSNR. The dynamic range and measurement floor are as relevant for the applicability and usefulness of a given measure for detection of change, especially in moderate-to-advanced glaucoma eyes. It has previously been shown that once a log unit of visual field damage is reached locally, all structural measures—including RNFL, BMO-MRW, and macular thickness—reach their measurement floor and cease to change after that point.^22,23,26,27^ In the clinical setting, utilizing multiple structural measures including the neuroretinal rim, RNFL, and macular thickness maximizes opportunities for early detection of worsening glaucomatous damage and it is possible that a combination of the structural measures may optimize detection of progression. This has been shown in many prior studies with regard to RNFL and macular measures.^26–28^

In summary, this study found that RNFL thickness measurements are more likely to demonstrate faster negative rates of change and to detect structural deterioration compared with BMO-MRW thickness in eyes with central or moderate-to-advanced glaucoma damage. Retinal nerve fiber layer RoC had a better LSNR than BMO-MRW globally and in more sectors, suggesting that RNFL thickness is superior to BMO-MRW thickness for monitoring change in such eyes.

## Supplementary Material

Legend for Supplementary Figures

Supplementary Figure 1

Supplementary Figure 2

## Figures and Tables

**TABLE 1.** Demographic and Clinical Characteristics of the 113 Study Eyes (113 Patients) at Baseline

Variable	
Age (Mean ± SD, years)	67.7 ± 8.4
Female, n (%)	70 (62%)
Number of scans (Median, (min, max))	10 (3–22)
Follow-up (Mean ± SD, years)	5.2 ± 1.3
Ethnicity, n (%)	
White	64 (57%)
Asian	24 (21%)
African American	13 (12%)
Hispanic	12 (11%)
Baseline global BMO-MRW (Mean ± SD, μm)	154.3 ±
Baseline global RNFL (Mean ± SD, μm)	52.3
Baseline BMO area (Mean ± SD, mm^2^)	60.3 ± 13.4
Baseline 24–2 visual field mean deviation	1.90 ± 0.50
(Mean ± SD, dB)	−9.2 ± 5.8

Abbreviations: BMO = Bruch membrane opening; BMO-MRW = Bruch membrane opening-minimum rim width; RNFL = retinal nerve fiber layer; SD = standard deviation.

**TABLE 2.** Estimated Population Average ± SD Thickness Measurements at Baseline (Intercepts) and Average Rates of Change (Slopes) of Retinal Nerve Fiber Layer (RNFL) and Bruch Membrane Opening-Minimum Rim Width (BMO-MRW) Measures Derived From Linear Mixed Models (LMM)

	Estimated Population Intercept (SE) ± SD (SE) μm	Estimated Population RoC (SE) ± SD (SE) μm/year
**RNFL**		
Global	62.8 (1.30) ± 13.9 (0.93)	−0.64 (0.08) ± 0.75 (0.06)
Temporal	50.7 (1.26) ± 13.4 (0.90)	−0.42 (0.08) ± 0.74 (0.07)
Superotemporal	74.8 (2.71) ± 28.8 (1.93)	−0.60 (0.16) ± 1.55 (0.12)
Inferotemporal	68.8 (2.80) ± 29.7 (2.0)	−0.90 (0.14) ± 1.25 (0.11)
Nasal	58.7 (1.47) ± 15.6 (1.06)	−0.50 (0.10) ± 0.82 (0.09)
Superonasal	75.1 (2.32) ± 24.5 (1.66)	−0.85 (0.12) ± 1.07 (0.10)
Inferonasal	71.3 (2.18) ± 23.1 (1.56)	−0.91 (0.14) ± 1.2 (0.11)
**BMO-MRW**		
Global	159.6 (4.78) ± 50.7(3.42)	−1.10 (0.27) ± 2.39 (0.23)
Temporal	128.3 (4.63) ± 49.0 (3.31)	−1.41 (0.26) ± 2.26 (0.24)
Superotemporal	141.1 (7.06) ± 74.7 (5.04)	−1.27 (0.37) ± 3.05 (0.33)
Inferotemporal	118.9 (5.57) ± 58.7 (3.99)	−1.18 (0.38) ± 3.12 (0.35)
Nasal	193.7 (6.55) ± 69.2 (4.68)	−0.82 (0.44) ± 3.98 (0.36)
Superonasal	178.4 (7.17) ± 75.7 (5.13)	−1.39 (0.42) ± 3.32 (0.38)
Inferonasal	176.7 (6.64) ± 70.2 (4.75)	−0.72 (0.40) ± 3.31 (0.35)

Averaged estimates for intercept and slopes are presented along with their corresponding standard error (SE) statistics.

**TABLE 3.** Proportion of Significantly Worsening and Improving Rates of Change for Retinal Nerve Fiber Layer (RNFL) and Bruch Membrane Opening-Minimum Rim Width (BMO-MRW) From Simple Linear Regression With Permutation Analyses

	RNFL		BMO-MRW	
	Proportion of Worsening Rates (%)	Proportion of Improving Rates (%)	Proportion of Worsening Rates (%)	Proportion of Improving Rates (%)
Global	54	5	43	9
Temporal	43	7	35	4
Superotemporal	35	5	35	8
Inferotemporal	38*	5	26*	12
Nasal	40	7	35	8
Superonasal	36	3	33	10
Inferonasal	50**	3	25**	7

* *P* = .02; McNemar’s test, RNFL had a higher proportion of significant rates of change

** *P* < .001; McNemar’s test, RNFL had a higher proportion of significant rates of change.

## Abbreviations:

OCT: optical coherence tomography
RoC: rates of change
RNFL: retinal nerve fiber layer
BMO-MRW: Bruch membrane opening-based minimum rim width
SLR: simple linear regression
LSNR: longitudinal signal-to-noise ratios
