# Supplementary material for: Comparing Rates of Change in Moderate to Advanced Glaucoma: Retinal Nerve Fiber Layer Versus Bruch Membrane Opening-Minimum Rim Width
Source: Am J Ophthalmol. Author manuscript; Available in PMC 2024 Feb 11. (PMC10859221; doi:10.1016/j.ajo.2023.05.003)
Supplement: Legend for Supplementary Figures [file NIHMS1959139-supplement-Legend_for_Supplementary_Figures.docx]

**Supplementary Figure 1.** Profile plots with Loess fit for global retinal nerve fiber layer (RNFL-G) thicknesses of the cohort patients. Each gray line displays the global thickness through the follow up duration for each eye. The thick blue line represents the Loess fit.

**Supplementary Figure 2.** Profile plots with Loess fit for global Bruch’s membrane Minimum rim width (MRW-G) thicknesses of the cohort patients. Each gray line displays the global thickness through the follow up duration for each eye. The thick blue line represents the Loess fit.
